# Onlay Uncemented Lateralized Reverse Shoulder Arthroplasty for Fracture Sequelae Type 1 with Valgus/Varus Malunion: Deltoid Lengthening and Outcomes

**DOI:** 10.3390/jcm9103190

**Published:** 2020-10-01

**Authors:** Alfonso Maria Romano, Adriano Braile, Pasquale Casillo, Guglielmo Nastrucci, Massimiliano Susanna, Angelo Di Giunta, Francesco Ascione

**Affiliations:** 1 Orthopedics and Sport Medicine Unit, Campolongo Hospital, 84025 Salerno, Italy; alfonso.maria.romano@gmail.com (A.M.R.); pasquale.casillo87@gmail.com (P.C.); guglielmonastrucci@hotmail.it (G.N.); 2 Department of Orthopaedic and Trauma Surgery, Ospedale Buon Consiglio Fatebenefratelli, 80123 Naples, Italy; 3 Dipartimento Multidisciplinare di Specialità Medico-Chirurgiche ed Odontoiatriche, Università degli Studi della Campania “Luigi Vanvitelli”, 80138 Naples, Italy; adriano.braile@hotmail.it; 4 Orthopedic and Traumatology Unit, San Donà di Piave Hospital, 30027 Venice, Italy; massimiliano.susanna@gmail.com; 5 Orthopaedic Division of Policlinico ‘G.B. Morgagni’, 95100 Catania, Italy; adigiunta@yahoo.com

**Keywords:** reverse shoulder prosthesis, fracture sequelae, post-traumatic arthritis, onlay lateralized uncemented stem, complications, deltoid wrapping, humerus fracture, tuberosities

## Abstract

The successful treatment of proximal humeral fractures remains challenging for shoulder surgeons, and failure rates are high, regardless of initial treatment. This study aimed to analyze the clinical and radiographic midterm results of onlay lateralized cementless stem reverse shoulder arthroplasty (RSA) in patients with valgus/varus malunion proximal humerus fracture sequelae without metaphyseal osteotomy. We retrospectively studied 35 cases with the diagnosis of fracture sequelae of the proximal part of the humerus with valgus/varus malunion. The mean duration of follow-up was 4.6 years (range, 2 to 7 years), and the mean time between fracture and arthroplasty was 6 years (1 to 32 years). Seventeen patients (48.6%) had initially been treated nonoperatively. The Constant score (CS), active range of motion, and radiographs of the affected shoulders, as well as the acromion to greater tuberosity (AGT) distance and deltoid length (DL), were analyzed before surgery and at their latest follow-up. A total of thirty-three patients (94.3%) rated their outcome as very good or good. Mean CS, forward flexion, and external rotation improved significantly (*p* < 0.0001), as did internal rotation and pain (*p* < 0.05). AGT distance significantly increased postoperatively from 14.7 to 43.3 mm, as did DL from 143 to 170 mm (*p* < 0.05). There was no correlation between the outcomes and valgus/varus deformity, previous surgeries, or AGT distance/DL. A total of four complications occurred (11.4%): two dislocations were detected (5.7%) and successfully revised with a longer cemented stem. Onlay lateralized uncemented stem RSA improves clinical outcomes and decreases complications when treating valgus/varus malunion fracture sequelae, avoiding intraoperative technical challenges, such as tuberosities osteotomy conscious of bone loss and proper deltoid tensioning.

## 1. Introduction

The successful treatment of complex humeral fractures remains challenging for shoulder surgeons, and failure rates are high, regardless of initial treatment. Soft tissue damage, scarring and contracture, tuberosity and shaft bone deformity, malunion, non-union and loss, and humeral head avascular necrosis (caused by the fracture or previous surgery) are suspected to be responsible for massive functional impairment of the affected shoulder joint and rotator cuff. These heterogeneous fracture sequelae are the sum of failed conservative treatment, failed internal fixation, and failed hemiarthroplasty.

Boileau [1,2] classified fracture sequelae of the proximal part of the humerus in four different pathologies. Subsequently, Moineau [3] divided Type 1 sequelae into four groups (Table 1).

The therapeutic choice of anatomical shoulder arthroplasty (TSA) or reverse shoulder arthroplasty (RSA) for fracture sequelae of the proximal humerus must initially be made on the basis of the Boileau classification [1,2,3] in an attempt to avoid secondary revision when possible since unsatisfactory results and high rates of complication for type 3 and 4 lesions have been reported after TSA. In patients in whom conservative treatment has failed, TSA showed promising midterm outcomes [1,2,3,4]. However, in patients with varus malunion and fatty infiltration of the rotator cuff [3], results were worse.

Restoring the anatomy of the proximal humerus and the congruity of the joint through anatomical arthroplasty can be difficult, especially in varus configurations. In proximal humerus valgus/varus malunion, TSA is not suitable, and RSA may provide an alternative [5]. Where a prosthetic replacement is possible without an osteotomy, surgeons should accept the distorted anatomy of the proximal humerus and adapt the prosthesis and their technique to the modified anatomy.

To our knowledge, neither a focus on the results of new design RSA (onlay, lateralized, and uncemented stems) in fracture sequelae without metaphyseal osteotomy nor a study restricted to valgus/varus malunion sequelae have appeared in the literature.

The aim of the present study was to analyze the clinical and radiographic midterm results of onlay lateralized cementless stem RSA in patients with type 1C and 1D proximal humerus fracture sequelae. We hypothesized that satisfactory functional results could be achieved while maintaining complication and revision rates manageable.

## 2. Material and Methods

Institutional review board approval was obtained to conduct a retrospective analysis of patients who had undergone RSA at the participating institutions.

Inclusion criteria were the diagnosis of type 1C and 1D sequelae of a fracture of the proximal humerus, according to Moineau [3], treatment with RSA, and a minimum follow-up of two years. Exclusion criteria were previous infection involving the affected shoulder, non-union, or previous arthroplasty of the shoulder. The results were retrospectively analyzed from prospectively gathered databases. Between 2012 and 2017, 42 patients were identified. Three were lost due to follow-up, and one died without adequate postoperative data, leaving 35 patients.

There were ten males and twenty-five were females. The dominant arm was affected in 31 patients. Their mean age at the time of arthroplasty was 71 years (54 to 87 years). The mean duration of follow-up was 4.6 years (range, 2 to 7 years). The mean time between fracture and arthroplasty was 6 years (1 to 32 years). Seventeen patients (48.6%) had been initially treated nonoperatively, and eighteen had undergone open reduction and internal fixation (ORIF), involving a plate in eight (22.9%), intramedullary nail in two (5.7%), and Kirschner wires percutaneous pinning (K-wires) in eight (22.9%). The hardware had been removed in separate prior procedures in all patients. Twenty-two shoulders had a preoperative valgus deformity, and thirteen a varus deformity.

### 2.1. Surgical Technique

Surgical technique and postoperative patient management have been previously described in detail [6].

Three senior shoulder surgeons (AMR, ADG and MS) implanted RSAs using the Equinoxe Shoulder System (Exactech Inc., Bloomington, MN, USA).

A deltopectoral approach was used in all cases. A subscapularis (SSC) tendon tenotomy was performed to access the joint. When possible (not excessive tissues and/or joint strain and acceptable tendon quality), the tenotomy was repaired with tendon-to-tendon sutures (28 RSAs had no SSC repair). The long head of the biceps was tenodesed to the pectoralis major when present.

The humeral canal was sized and then compacted until rotational stability of the trials was achieved. None required a cemented stem. Glenosphere diameters were 38 mm for females and 42 mm for males. No metaphysis/tuberosity osteotomies were associated with RSA implant.

### 2.2. Clinical Evaluation

Patient pre- and post-operative active range of motion (ROM) assessments including forward flexion (FF), external rotation (ER) with the arm at the side, internal rotation with the level of the thumb on the spine (IR), the Constant–Murley Score (CS) [7], perioperative data (implant sizes, intraoperative complications), and postoperative complications were collected. Severe stiffness, defined by external rotation of < 0°, was present preoperatively in 20 patients (57.1%).

The patients were asked to rate their outcome as very good, good, satisfactory, or unsatisfactory. The postoperative clinical evaluation was performed by two independent examiners not involved in the surgery (FA and PC).

### 2.3. Radiographic Evaluation

Standardized pre- and post-operative radiographic films, including a true anterior-posterior (AP) view with three different rotations of the arm (internal, external, and neutral) and the scapular Y-view, and preoperative CT scans were obtained.

The radiographic parameters evaluated before and after surgery were the acromion to greater tuberosity distance (AGT) and deltoid length (DL) on a true anteroposterior view in neutral rotation [8] for each patient. This was used to assess postoperative deltoid lengthening rather than that described by Ladermann [9] because, in this particular etiology, the method is more relevant to postoperative deltoid wrapping [10].

**Figure 1:**
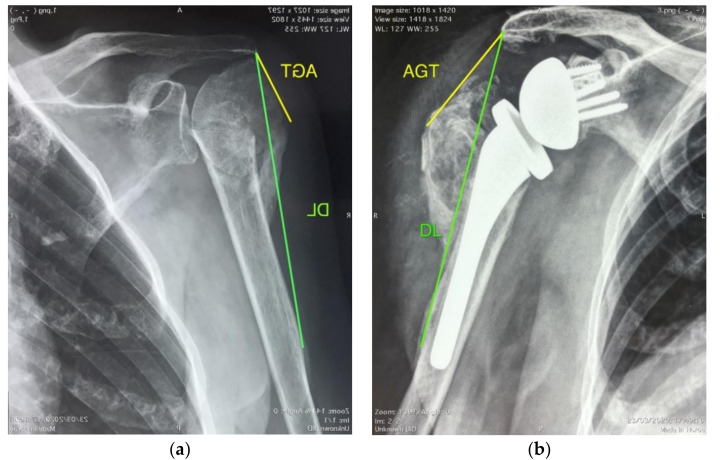
Radiographic acromion to greater tuberosity distance (AGT) and deltoid length (DL); (**a**) preoperative measurement; (**b**) postoperative measurement.

Preoperative AGT distance was measured from the most inferolateral aspect of the acromion to the most superolateral aspect of the humerus, which was the greater tuberosity. Postoperative AGT distance was measured from the inferolateral tip of the acromion to the most superolateral aspect of the humerus or humeral component. Deltoid length was measured from the inferolateral tip of the acromion to the deltoid tuberosity on the humerus (Figure 1). The position of the greater tuberosity relative to the humeral diaphysis was used to define varus or valgus malunion of the proximal humerus, as described by Moineau [3]. CT images were analyzed for cuff muscle fatty infiltration grade.

Radiological examination included analysis of AP and Y-views at the final follow-up, documenting the scapular notching grade, the inferior spur at the region of insertion of triceps, signs of loosening, and/or signs of ossification and osteolysis around the components.

### 2.4. Statistical Analysis

Statistical analysis was performed using the Social Science Statistics collaborative website (http://www.socscistatistics.com). Differences between preoperative and final follow-up data were compared using the Student *t*-test for paired data; the Fisher test or the Chi-square test were used to identify relationships between variables. Significance was set at *p* < 0.05.

## 3. Results

A total of twenty-one patients (60%) rated their outcome as very good, twelve (34.3%) as good, and two (5.7%) as unsatisfactory.

### 3.1. Clinical

The mean Constant score increased from 23.5 points (range, 5 to 40 points) preoperatively to 66.7 points (14 to 87 points) postoperatively. The mean forward elevation of the shoulder increased from 56.9° (range, 30° to 120°) preoperatively to 134.8° (45° to 170°) postoperatively. The clinical outcomes are shown in Table 2.

The average Constant score, forward flexion, and external rotation improved significantly (all *p* < 0.0001), as well as the internal rotation and pain (both *p* < 0.05).

The mean postoperative Constant scores (*p* = 0.10) and range of motion were not significantly different in patients with preoperative stiffness compared with those without. No differences were found between patients who were initially treated nonoperatively and those who were treated surgically with regard to the Constant score (*p* = 0.30), shoulder flexion (*p* = 0.54), external rotation (*p* = 0.38), and internal rotation (*p* = 0.22).

There was no difference in the mean postoperative Constant score between those with a valgus deformity and those with a varus sequela (*p* = 0.72).

### 3.2. Radiographic

There was no radiological evidence of loosening of either component. There was inferior scapular notching of grade 1 in 4 patients (11.4%); no inferior spurs were found. There was no evidence of osteolysis or progressive ossification.

AGT distance increased significantly postoperatively from 14.7 ± 13 to 43.3 ± 12 mm as well as DL from 143.1 ± 21.3 to 170 ± 11.6 mm (both *p* < 0.05). No significant differences between clinical outcomes (ROM and CS) and radiographic measurements of AGT distance or DL were observed.

### 3.3. Complications

**Figure 2:**
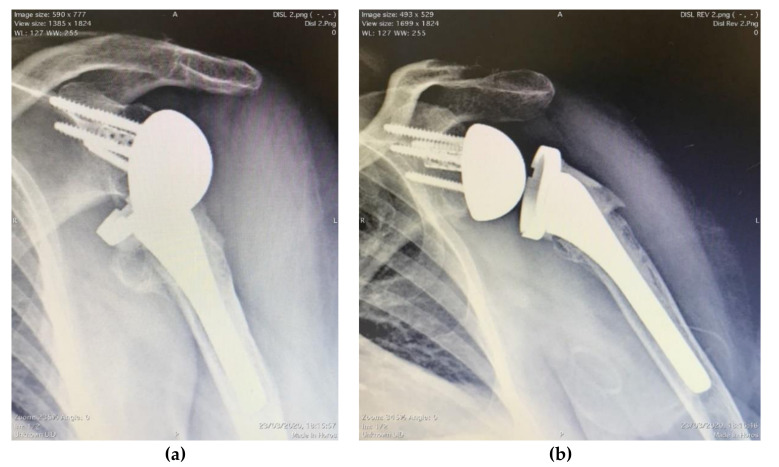
A case of dislocated arthroplasty (**a**), successfully treated through a revision of the implanted stem, replaced with a longer cemented one (**b**).

A total of four complications occurred (11.4%). A traumatic fracture of the acromion (2.9%) was detected radiographically in one patient, three years postoperatively, with no clinical consequences. One patient developed a deep infection (2.9%) caused by *Cutibacterium acnes* three months postoperatively: a polyethylene insert replacement and debridement successfully eradicated the infection. Two dislocations were detected (5.7%); the subscapularis tendon was repaired in both previous RSA implants. The first patient had a proximal humeral fracture previously treated by K-wires pinning: after the first prosthesis dislocation, a closed reduction and one-month sling immobilization were attempted; after a second dislocation, an RSA revision was performed with a long cemented stem, higher humeral adapter, and thicker insert, in consideration of the poor humeral stability and deltoid tension; after a further dislocation, a hemiarthroplasty was implanted. The second dislocated arthroplasty was treated through a revision of the implanted stem and was substituted with a longer cemented one (Figure 2). There was no glenoid radiolucency. Humeral nonprogressive radiolucency was found in 5 cases. No peripheral neuropathies were recorded.

## 4. Discussion

From a review of shoulder arthroplasty literature, it can be concluded that satisfactory results are expected in 15 to 72% of shoulder fracture sequelae cases, with pain relief in more than 85%. Motion is usually limited, with an active anterior elevation of around 110° and an active external rotation of around 10°. Complication and revision rates are usually higher in these cases than in other etiologies (from 20 to 48% and from 3.5 to 35%, respectively) [1,2,3,5,6,11,12,13,14,15,16,17,18,19,20,21,22,23,24,25].

Wall (11) reported the results of 33 cases of RSA for post-traumatic sequelae in a larger series of 191 arthroplasties. Results were inferior (CS of 53 points) to RSA performed for cuff tear arthropathy, massive rotator cuff tears, or osteoarthritis with cuff tears.

Raiss [5,19,20,21] studied the topic in detail, reporting satisfying functional results after RSA for every fracture sequelae pattern. Those for type 2 and 3 lesions were less promising, as there were high rates of complication: 32% for type 2 and 41% for type 3. The results in those with a severe malunion of the tuberosities (type 4) were clinically encouraging, with a rate of complication of 9.5%, and nearly 90% of patients being either satisfied or very satisfied with the outcome (mean follow-up, four years).

In type 1 sequelae, the mean CS improved from 25 points (5 to 47 points) preoperatively to 57 points (15 to 81 points) postoperatively. The mean forward elevation of the shoulder increased from 73° (10° to 130°) preoperatively to 117° (15° to 170°) postoperatively [5].

It was found that preoperative rotator cuff dysfunction leads to significantly lower postoperative CS. However, previous surgery and configuration of malunion of the proximal humerus, even in varus or valgus, did not influence the outcome. These results, along with others reported in the literature, are comparable to the findings of the current study, only slightly superior to Grammont RSA studies [1,2,5,6,11,12,13,14,15,17,18,19,20,21,22,23,24,25,26,27]. Range of motion and CS significantly improved after RSA, as well as the reported pain relief, reporting 33 patients out of 35 (94.3%) were satisfied or very satisfied at the final follow-up (Table 2). The present study recorded no significant difference in clinical outcomes between valgus deformity cases and those with a varus sequelae, in contrast with Moineau’s negative prognostic factors [3].

It is often impossible to achieve the anatomic reconstruction of the humerus with conventional stemmed shoulder replacements, and several factors seem to influence final results. The outcome is better after initial conservative treatment (when compared to initial surgical treatment) in cases without any distortion of the tuberosities and post-traumatic arthritis with avascular necrosis (compared to post-traumatic arthritis with non-union, humeral head-metaphysis defect, and/or malunion). A greater tuberosity osteotomy is most likely responsible for the poor, unpredictable results and increased complications after shoulder replacement arthroplasty in this context [1,2,3,4,12,17]. Therefore, our study dealt with an onlay lateralized stem consecutive arthroplasty without a tuberosity osteotomy, showing acceptable ouctomes and complications.

The constraints and torsional forces of Grammont-type RSA implants can lead to changes on the humeral side, such as osteolysis, osteopenia, and the development of medial and lateral cortical bone narrowing [16,28,29] due to the medialization of the implant protecting the glenoid from stress. This is greater on the humerus, leading to recurrent dislocations, stem loosening, prosthetic dissociation, and humeral component unscrewing and consequentially to various types of RSA failures [11,16]. Ascione [28] reported 3.3% of humeral complications after RSA in a long-term follow-up multicenter study of 1035 Grammont arthroplasties: complications were more common following fracture sequelae (or difficult cases such as revision for failed arthroplasty), and postoperative humeral fractures/humeral loosening were exclusive relevance of cemented stems, that were not needed to implant in our cases. No humeral stem-related problems and/or reinterventions were reported in our series (fractures, loosening/migration, and disassembly), despite what the literature generally attributes to component complications (1.5–10%) and reinterventions (12–27%) [2,16,28,30,31].

In recent years, alternative stemless or short-stemmed implants with metaphyseal fixation, with an onlay design, have been developed specifically for fracture sequelae to position the prosthetic head independently of the humeral shaft [14,32,33,34]. Only a limited number of reports on the outcomes of treatment using these new implants have been published but with promising early results.

The complications reported in RSA after fracture sequelae included humeral fracture, nerve palsy, dislocation, and loosening. Our complication rate was 11.4%, in particular, dislocation (5.7%) and revision (5.7%). Data were consistent with the literature, with a relevant improvement in revision surgeries: all reported fracture sequelae complication rates ranged from 16 to 41%, and revision rates ranged from 11 to 41% [2,3,18,23,24,35]. Interestingly, RSA instability appeared to be the main complication in fracture sequelae case series, ranging from 9.5 to 17%. In fact, a significant increase in rotational micromotion with proximal bone loss, such as fracture sequelae cases, was demonstrated [5,36].

The scapular notching rate was reported to be greater than 50% in most series [11,13,15,16,23,37]. This current study recorded 11.4% of grade 1 scapular notching, despite the specific etiology, but was consistent with the onlay lateralized RSA case series [38,39,40].

Greiner [22] evaluated prognostic factors after RSA in fracture sequelae, highlighting that loss of pretensioning of the deltoid or weakening of the deltoid insertion in bone loss (especially larger than 3 cm), in addition to previous operations and an incompetent teres minor, may be contributing factors in the poorer results. This was confirmed by two dislocation cases in the present series, both with humeral proximal bone loss and insufficient deltoid tensioning, subsequently successfully revised using longer components. Sabesan [8] found that deltoid lengthening and increase in AGT distance negatively affected postoperative forward elevation, whereas Ladermann [9] found no correlation between deltoid lengthening and improvements in the range of motion, but significant improvement in patients’ postoperative forward flexion.

The aforementioned studies discussed Grammont inlay medialized RSA. Werner [41] studied 56 lateralized onlay stem RSAs, and found a correlation between arm lengthening exceeding 2.5 cm and decrease in anterior elevation, and a relationship between arm lengthening averaging 2.2 cm and increased outcomes, concluding that 1 to 2.5 cm of arm lengthening is the best compromise on the postoperative range of motion. In our series, we found no significant differences between the clinical outcomes and AGT distance or DL, despite a mean increase of 2.9 cm and 2.7 cm, respectively.

Nevertheless, a defect of tensioning of deltoid can lead to RSA instability, especially in a particular pathology, such as post-traumatic osteoarthritis, with heterogeneous former treatments of fracture and different periods elapsed to RSA implant (1 to 32 years in the present series), in which deltoid has become hypotrophic.

### Limitations

Shortcomings inherent to a nonrandomized and retrospective study were the major limitations. In addition, the retrospective design may have resulted in an under-reporting of complications, and there was an inadequate follow-up to assess long-term clinical and radiographic outcomes. The rate of complication or need for reoperation surgeries may also increase over time.

However, the study was conducted over a short period, and selected surgical indications, based on clinical and radiological assessments, surgical techniques, and rehabilitation protocols were standardized for both surgeons. Still, a two-surgeon study might remain a bias.

Despite the relatively small number of cases in the current study, the results were quite promising.

The cohort of patients was small but consistent with other studies of RSA for fracture sequelae. Moreover, to our knowledge, this is the largest series available reporting on the results of type 1 sequelae of a fracture of the proximal humerus treated only by onlay lateralized RSA.

## 5. Conclusions

In conclusion, RSA is an effective form of treatment for patients with type 1 sequelae of a fracture of the proximal humerus, in association with valgus/varus malunion, leading to high rates of satisfaction. Deltoid lengthening does not influence the outcome, but a defect in deltoid tensioning and humeral proximal bone loss can lead to instability, which remains the most worrying complication in this etiology.

Onlay lateralized stem improves clinical outcomes, reduces complications, and minimizes intraoperative technical challenges, such as tuberosities osteotomy; the need for revision is dramatically reduced by onlay lateralized uncemented RSA, provided that the metaphyseal bone stock is preserved.

## Figures and Tables

**Table 1:** Boileau and Moineau classification of humerus proximal part fracture sequelae.

Fracture Sequelae		
*Intracapsular*	Type 1	
1a		isolated post-traumatic osteonecrosis of the humeral head without tuberosity malunion
1b		isolated post-traumatic osteoarthritis without osteonecrosis or tuberosity malunion
1c		proximal humeral deformity with valgus malunion secondary to a valgus impacted fracture
1d		varus malunion secondary to a varus impacted fracture
	Type 2	related to locked dislocation or fracture-dislocation
*Extracapsular*	Type 3	surgical neck non-union
	Type 4	severe tuberosity malunion

**Table 2:** Clinical outcomes.

	Preoperative	Postoperative	Δ	*p*
**FF (deg)**	56.9 ± 22.4	134.8 ± 29.8	77.9	<0.0001
**ER (deg)**	−4.5 ± 18	12.9 ± 15.1	17.4	<0.0001
**IR (0–10)**	2.1 ± 2	5.3 ± 2.3	3.4	<0.05
**CS Pain/15**	3.7 ± 1.8	12.2 ± 2.6	8.5	<0.05
**CS%**	23.5 ± 14.3	66.7 ± 19.7	43.2	<0.0001

FF: forward flexion; ER: external rotation with elbow at side; IR: internal rotation; level of the thumb on the spine; CS: Constant score.
